# The evolutionary dynamics of major regulators for sexual development among Hymenoptera species

**DOI:** 10.3389/fgene.2015.00124

**Published:** 2015-04-10

**Authors:** Matthias Biewer, Francisca Schlesinger, Martin Hasselmann

**Affiliations:** ^1^Population Genetics of Social Insects, Institute of Genetics, University of CologneCologne, Germany; ^2^Livestock Population Genomics Group, Institute of Animal Science, University of HohenheimStuttgart, Germany; ^3^Institute of Bee ResearchHohen Neuendorf, Germany

**Keywords:** gene duplications, sex determination, adaptive evolution, regulatory changes, pathway evolution

## Abstract

All hymenopteran species, such as bees, wasps and ants, are characterized by the common principle of haplodiploid sex determination in which haploid males arise from unfertilized eggs and females from fertilized eggs. The underlying molecular mechanism has been studied in detail in the western honey bee *Apis mellifera*, in which the gene *complementary sex determiner* (*csd*) acts as primary signal of the sex determining pathway, initiating female development by *csd*-heterozygotes. *Csd* arose from gene duplication of the *feminizer* (*fem*) gene, a *transformer* (*tra*) ortholog, and mediates in conjunction with *transformer2* (*tra2*) sex-specific splicing of *fem*. Comparative molecular analyses identified *fem*/*tra* and its downstream target *doublesex* (*dsx*) as conserved unit within the sex determining pathway of holometabolous insects. In this study, we aim to examine evolutionary differences among these key regulators. Our main hypothesis is that sex determining key regulators in Hymenoptera species show signs of coevolution within single phylogenetic lineages. We take advantage of several newly sequenced genomes of bee species to test this hypothesis using bioinformatic approaches. We found evidences that duplications of *fem* are restricted to certain bee lineages and notable amino acid differences of *tra2* between *Apis* and non-*Apis* species propose structural changes in Tra2 protein affecting co-regulatory function on target genes. These findings may help to gain deeper insights into the ancestral mode of hymenopteran sex determination and support the common view of the remarkable evolutionary flexibility in this regulatory pathway.

## Introduction

Understanding the evolution of biological pathways and the driving processes shaping them still belongs to the central questions in biology. Studying genetic networks and their underlying selectional and developmental processes can provide important insights into the divers evolutionary trajectories of molecules between species (Pires-da Silva and Sommer, 2003; Wilkins, 2007; Fani and Fondi, 2009; Davidson, 2010; Peter and Davidson, 2011). As one common process, gene duplication has been identified to play a key role in providing novel or modified gene functions resulting from various forms of selection acting on the paralogous copies (Innan and Kondrashov, 2010). Following the model of neofunctionalization, a paralogous copy may acquire a novel function not present in the gene from which it arose. Besides positive selection promoting the fixation of advantageous mutations in this copy, exon or domain shuffling may further contribute to the evolution of a neofunctionalized gene. Among others, the well-established duplication-degeneration-complementation (DDC) model provides a basis for the evolution of an modified (subfunctionalized) function in the paralogous gene (Force et al., 2005).

The sex determination pathway in honey bees constitutes a well-studied example, in which gene duplication has been identified to play a major role in its evolutionary history (Hasselmann et al., 2008). Common for all hymenopteran species (ants, wasps, and bees) is the principle of haplodiploidy in which males are haploid and develop from unfertilized eggs, whereas females are diploid and arise from fertilized eggs (Bull, 1983). The underlying molecular signals and regulatory key genes involved in the sex determination pathway have been studied in greater detail for only two hymenopteran species, the parasitic wasp *Nasonia vitripennis* (Beukeboom et al., 2007; Verhulst et al., 2013; van de Zande and Verhulst, 2014) and the western European honey bee *Apis mellifera* (Beye et al., 2003; Hasselmann and Beye, 2004; Hasselmann et al., 2008; Gempe et al., 2009) with an estimated divergence time of about 170 million years ago (Werren et al., 2010).

With the now available new genomes of bee species covering a divergence time of about 100 million years, we are closing the so far existing gap between *Apis* and *Nasonia*. Consequently, we can now study the evolution of the sex determination pathway and the driving forces shaping key components on a refined scale. Thus, one of the obvious questions is whether lineage specific events such as gene duplications can be observed to affect key regulator coevolution. Within the sex determination pathway of insects, a conserved unit of genes has been identified, giving rise to a transductional core downstream of the primary signal (Bopp et al., 2014) that transmits the information of the primary signal and releases male/female specific developmental regulatory signals to a variety of target genes. We hypothesize that the core unit of sex determining genes is relative conserved in all Hymenopterans; however, evolutionary processes may have shaped these genes and the additional cofactors lineage specifically.

In the honeybee, the primary signal is the gene *complementary sex determiner* (*csd*), which arose from gene duplication of its copy *feminizer* (*fem*) (Beye et al., 2003; Hasselmann et al., 2008). The molecular decision of male or female development is mediated by a multiallelic system of protein-protein interaction, in which a heterozygous conformation leads to female development, while homo- and hemizygotes develop into males. The evolutionary history of the paralogous genes has been shaped by contrasting forms of selection in *Apis*: after the duplication, *csd* experienced strong positive selection, following the model of neofunctionalization, whereas *fem* evolved under strong purifying selection (Hasselmann et al., 2010). The formation of specific protein regions such as a hypervariable region (HVR) and a protein-interacting coiled-coil motif are important for the rise and function of *csd*-alleles. Molecular functional analysis provided evidence for sex-specific splicing of *fem*, initiated by the allelic state of *csd*, in which heterozygote *csd* lead to female-specific *fem*-mRNA splicing. Acting as binary switch gene, *fem* transcripts are maintained and enhanced by an autoregulatory feedback loop of the Fem protein (Hasselmann et al., 2008; Gempe et al., 2009). This serine-arginine (SR) rich protein and its ortholog *transformer* (*tra*) are differentially spliced, either to a female functional or to a male nonfunctional isoform, as found for other insect species (Butler et al., 1986; Pane et al., 2002; Sarno et al., 2010; Verhulst et al., 2010). The processing of sex-specific information by the *fem*/*tra* gene is conserved in these insects and the sex determining pathway converged at this level (Gempe et al., 2009).

The absence of an RNA recognition motif (RRM) domain in *Apis fem* requires a cofactor protein for RNA binding to mediate the sex-specific splicing process. It has been shown by Nissen et al. (2012) that the Transformer2 (Tra2) protein in conjunction with the Csd protein transmit the sex-specific splicing of *fem*-mRNA. Tra2 is evolutionary conserved among insects and characterized by a single, 80–90 amino acid long RRM domain, flanked by two SR domains. Two sequence elements (RNP1 and RNP2) have been shown to be directly involved in RNA recognition. With this ability to recognize RNA motifs, Tra2 facilitates the *fem/tra* autoregulatory splicing loop, which can be found in other insect species, except *Drosophila* (Gempe et al., 2009; Salvemini et al., 2009; Hediger et al., 2010; Sarno et al., 2010).

The female-specific active Fem (Tra)/Tra2 complex regulates the differential splicing of the downstream target for sex-specific development, *doublesex* (*dsx*). The gene *doublesex* (*dsx*) represents the key gene in sex determination of insects as the most downstream component of the pathway regulating sex-specific phenotypes (Burtis and Baker, 1989; Cline and Meyer, 1996). Acting as transcription factor, *dsx* encodes a protein with a zinc-finger DNA-binding domain (DM domain). In all insect species studied so far, gene structure and pattern of sex-specific splicing is generally conserved (Cho et al., 2007). Female and male transcripts consists of two oligomerization domains (OD1 and OD2) harboring DNA and protein interaction functions. The use of different splice sites at the C-terminal region results in OD2 sequence variation that alters the female- and male-specific regulation of target genes, which regulates the sex-specific splicing of pre-mRNA into male or female isoforms for the particular development as an essential transductional core of the pathway.

Among hymenopteran non-*Apis* species, the molecular basis of sex determination is best understood for the phylogenetically most basal parasitic wasp *Nasonia*, in which an alternative mode of haplodiploid sex determination evolved (Verhulst et al., 2013). Similar to what is known for many other dipteran insects, *transformer* mRNA of *Nasonia vitripennis* (*Nvtra* mRNA) is maternally provided to all eggs, however only in fertilized eggs *Nvtra* transcription can initiate and maintain female *Nvtra* mRNA by an autoregulatory feedback loop. In unfertilized eggs, the maternally provided genome induces low level of *Nvtra* expression, leading to the hypothesis of genomic imprinting as sex determination mechanism (Verhulst et al., 2010). Recent findings indicate that alleles of an trans-acting factor *(womanizer)*, likely to be maternally provided may have been recruited as novel component in the sex determining pathway (Verhulst et al., 2013).

There is increasing evidence that the initial signals of sex determining pathways may evolve rapidly, contributing to the astonishing diversity of species. The underlying processes driving this rapid evolution may be gene duplications, accompanied by the gain of novel or modified function and changes in the selective regime under which the key genes evolve. In our study we provide evidence for the importance of instantaneously occurring events such as gene duplications and lineage specific mutations that affect key regulator coevolution within the sex determination pathway of hymenopteran species.

## Materials and methods

### Sequence data

Genome assemblies and annotations of recently sequenced bee species (Kapheim et al., in revision) were used to identify gene copies of interest (*feminizer*—*fem, transformer2*—*tra2, doublesex*—*dsx*), taking Amell vs. 4.5, OGS 3.2 as reference and using various blast parameters to avoid non-detection errors. Hidden Markov profile searches (Eddy, 1998) were performed to search specifically for *fem* paralogs in bee genomes using HMMer3 on protein (HMMsearch) and nucleotide (nHMMer) level (Eddy, 2009). Multiple sequence alignments were generated using MUSCLE (Edgar, 2004) and optimized manually. To reduce the loss of informative sites due to incomplete or misleading annotations, experimentally proven and publicly available data were used for some species and GenBank and OrthoDB entries were used for *fem* and paralogous copies, *tra2* and *dsx* sequences. The sequences used for our analyses for comparing functional and evolutionary relationships were retrieved from GenBank and OrthoDB. Accession numbers are given in the Supplementary Tables 1–3.

### Evolutionary analyses

Genealogies were reconstructed after applying Model Test (Posada and Crandall, 1998) on the dataset to determine the evolutionary substitution model that fitted the data best. The model with the lowest BIC (Bayesian Information Criterion) scores was considered best for describing the substitution pattern. Non-uniformity of evolutionary rates among sites was modeled by using a discrete Gamma distribution (+G) with 5 rate categories. Evolutionary trees were constructed using the maximum likelihood method (JTT model) implemented in MEGA6 (Tamura et al., 2013). Examination of exonic splicing regulatory elements (ESR) was performed on the ESR search website (http://esrsearch.tau.ac.il/) using the highest number of available parameters (Fairbrother et al., 2002). Further, analyses of conserved protein domains and protein function were performed with conserved domain search module (Marchler-Bauer et al., 2011) implemented on www.ncbi.nlm.nih.gov and InterPro (Hunter et al., 2012), http://www.ebi.ac.uk/interpro/). The program COIL (implemented online under: http://embnet.vital-it.ch/software/COILS_form.html) was used to search specifically for predicted coiled-coil regions. The COIL program compares the query sequence to a database of known coiled coils and derives a similarity score. The probability that the sequence will form a coiled-coil motif is obtained within the program by comparing the similarity score against the distribution of scores in globular and coiled-coil proteins. Sequence-based motifs were identified and analyzed using the MEME suite package (http://meme.nbcr.net/meme/, Bailey et al., 2009). The significance of the motif is determined by first finding the most statistically significant (low *E*-value) motifs. Motifs are shown as sequence logos, represented by position-specific probability matrices that specify the probability of each possible letter appearing at each possible position in an occurrence of the motif. Displayed as stacks of letters at each position in the motif, the total height of the stack is the “information content” of that position in the motif in bits.

## Results

### Diversification of *feminizer* gene duplicates

**Figure 1:**
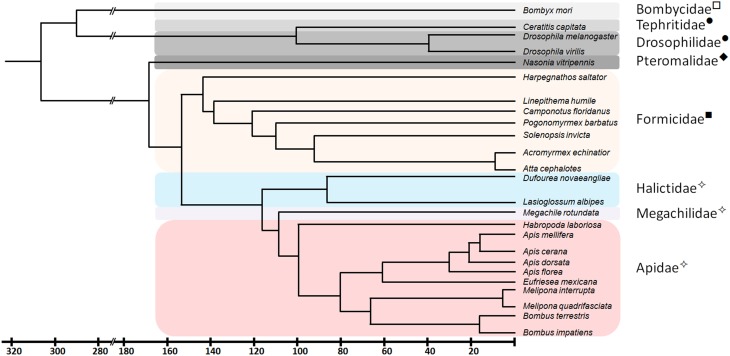
**Phylogenetic relationship and divergence time of species analyzed in this study**. Common names of the different insect families are marked: ^✧^ - Bees, ^■^ - Ants, ^♦^ - Wasps, ^•^ - Flies, and ^□^ - Moths. Redrawn from Grimaldi and Engel (2005), Gadau et al. (2012), Cardinal and Danforth (2013) and *Drosophila* genome database (www.flybase.org).

**Figure 2:**
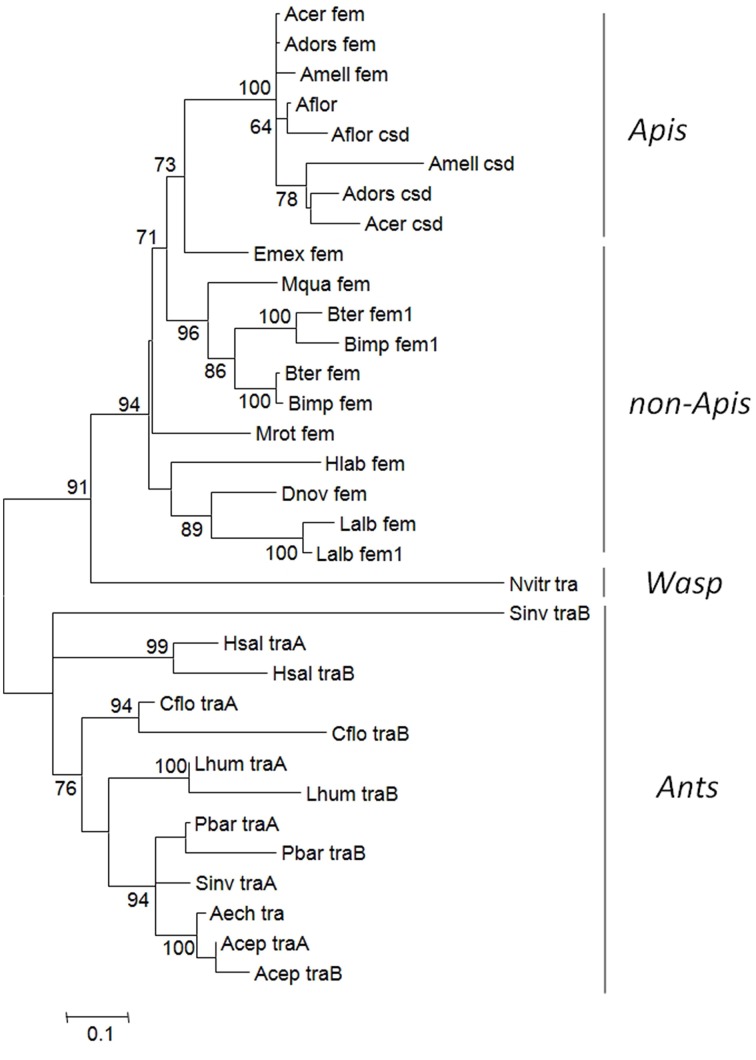
**Overview of the evolutionary relationship of the *fem* gene and copies (*fem1, csd, tra*) in social insect species**. The tree with the highest log likelihood was inferred by using the Maximum Likelihood method. The percentage of trees in which the associated taxa clustered together is shown next to the branches. Pairwise amino acid distances estimated using a JTT model using a discrete Gamma distribution was applied to model evolutionary rate differences among sites. All positions with less than 95% site coverage were eliminated. Abbreviation of species: Aech, *Acromyrmex echinatior;* Acep, *Atta cephalotes;* Acer, *Apis cerana*; Adors, *Apis dorsata*; Aflor, *Apis florea*; Amel, *Apis mellifera*; Bimp, *Bombus impatiens*; Bter, *Bombus terrestris*; Cflor, *Camponotus floridanus*; Dnov, *Dufourea novaeangliae*; Emex, *Eufriesea mexicana*; Hlab, *Habropoda laboriosa*; Hsal, *Harpegnathos saltator*; Lalb, *Lasioglossum albipes*; Lhum, *Linepithema humile*; Mquad, *Melipona quadrifasciata*; Mrot, *Megachile rotundata*; Nvitr, *Nasonia vitripennis;* Pbar, *Pogonomyrmex barbatus*; Sinv, *Solenopsis invicta*.

In a previous study (Kapheim et al., in revision), we have identified *fem* paralogs and orthologs of recently obtained genomic resources of bees representing different levels of social organization (Figure 1). Representative species were analyzed from Apini (the western European honey bee *Apis mellifera* and the dwarf honey bee *Apis florea*), Bombini (the buff-tailed bumble bees *Bombus terrestris* and *Bombus impatiens*), Euglossini (the orchid bee *Eufriesea mexicana*), Meliponini (*Melipona quadrifasciata*), Megachilini (the leafcutter bee *Megachile rotundata*) and Halictini (*Lasioglossum albipes* and *Habropoda laboriosa*). We noticed that the occurrence of *fem* duplications varies among different lineages in conjunction with varying signs of diversifying and negative selection. When including *transformer* (*tra*) orthologous sequences of seven ant species and one parasitic wasp, the sequences fall into two major clades, separating ant-*tra* from the remaining sequences (Figure 2). All genes share an arginine-serine rich and a proline rich domain, establishing these copies as strong candidates to be involved in protein interaction and splicing processes. For the paralogous genes *fem* and *csd* within the *Apis* lineage, evidence for both processes has been given by numerous functional studies (Hasselmann et al., 2008; Gempe et al., 2009; Nissen et al., 2012). In the bumble bee *Bombus terrestris*, for *fem* and its paralogous copy *fem1* several splice forms were identified (Biewer et al., in revision); in the stingless bee *Melipona interupta* the single copy *fem* gene is characterized by two splice forms (Brito et al., unpublished).

Here, we focus on the evolutionary dynamic of Fem proteins among bees using amino acid sequence motifs. We follow the hypothesis that characteristic motifs should be found in all bees harboring changes in species-specific paralogs of *fem*. These could hint to lineage-specific modifications of protein interaction in the sex determination pathways. Our hypothesis is supported by the previous study of Koch et al. (2014) showing the independent origin of *fem* paralogs in *Apis* and *Bombus* (and Ants) and thus different evolutionary fates, for which the multiallelic evolution of *csd* stands as one remarkable example (Hasselmann et al., 2008; Lechner et al., 2014).

**Figure 3:**
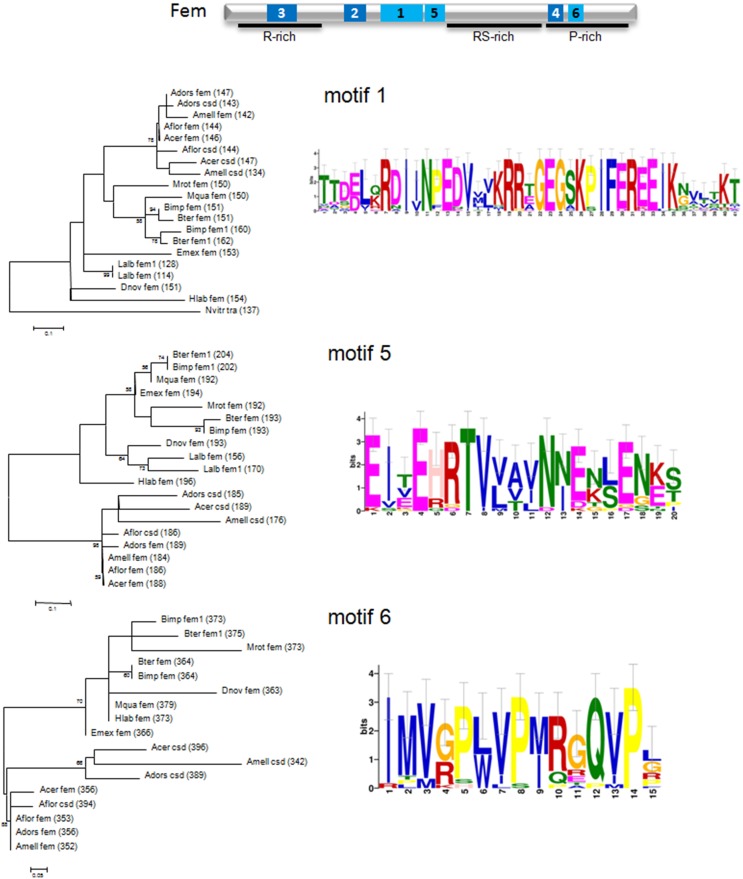
**Schematic view of the Fem protein, sequence logos of amino acid motifs and their phylogenetic signal of *fem* and paralogous copies**. Motifs are marked in pale blue (1, 5, 6) and dark blue (2, 3, 4), specified domains with a black line. Conserved motifs were identified using the MEME package (see Materials and Methods) and maximum likelihood trees represent amino acid per site divergence. Small-sample correction was applied and represented by error bars for each letter.

In order to test our hypothesis, we first evaluated the amino acid motifs in Fem protein sequences of bee species and the wasp *Nasonia vitripennis* using the MEME program package (see Materials and Methods). Six motifs with the best scoring *E*-values (*E*-values ranging from 1.0e^−488^ to 8.0e^−143^) were detected, represented by sequence logos (see Figure 3 and Supplementary Figure 1). The relative positions of these motifs in the protein are located in the N-terminal and C-terminal, as well as in-between regions of the protein (Supplementary Figure 2). Sequence logos illustrate the evolutionary conservation of several amino acids, the most prominent ones are Glutamic acid (E), Arginine (R), Lysine (K), Glutamine (G) and Proline (P), as well as variable positions, giving rise to species-specific divergence.

Next, we evaluated the phylogenetic signal for each motif by constructing genealogies based on the maximum likelihood algorithm. Amino acid divergence of *Apis* compared to non-*Apis* bees is most pronounced for motif 1, 5, and 6, resulting in three separated and clearly supported (78–98% bootstrap support) clusters. The sequence clustering is less obvious for motif 3 and an unresolved branching pattern results from motif 2 and 4. Interestingly, motif 5 locates in direct vicinity of the predicted coiled-coil (cc)-motif, identified to be specifically evolved in *csd* of *A. mellifera, A. cerana* and *A.dorsata* by positive selection of six non-synonymous changes (Hasselmann et al., 2008). No such cc-motif can be detected on the homolog positions in *A. florea csd* (Biewer et al., in revision) and in those of *fem* or its paralogs for other hymenopteran species (Supplementary Figure 3). However, we identified the presence of a cc-motif in the region of motif 3 for *A. florea csd* (Biewer et al., in revision) that coincides with an α and β sheet PLP-dependent transferase-like structure predicted by the SMART tool (http://smart.embl-heidelberg.de/) in non-*Apis* bees. We conclude that at least regions of motif 3 and 5 are candidates for having resulted from lineage-specific evolution in protein interaction processes associated with the sex determination pathway in bees.

### Lineage specific coevolution of *Transformer2*

Subsequently, we followed the hypothesis that coevolutionary signs should be detectable within the *tra2* gene as major co-regulator in the sex determining pathway, if the key regulator *fem* and (if present) its paralogous copies evolved with a modified function. Therefore we first aligned Tra2 protein sequences from orthologs of 10 bee species and three other insect species (*N. vitripennis, B. mori, D. melanogaster*) and focused on the RNA recognition motif (RRM), which is flanked by two SR rich regions. The RRM contains about 80 aa and forms a βαββαβ barrel motif, whereas on the third β sheet the two conserved elements RNP1 and RNP2 are located, known to be directly involved in the RNA recognition of *dsx* in *D. melanogaster* (Chandler et al., 1997), Figure 4. No amino acid changes between bee species exist in RNP2 whereas the remaining part of the RRM show pronounced differences among the species. Two observations are of particular interest: First, all non-bee species compared to the bee species show numerous amino acid changes, ranging from 9 aa (*Nvit*) to 28 aa (*Dmel*) which reflects their phylogenetic distance. Second, within the bee species, the *Apis* species *A. mellifera* and *A. florea* are consistently different for 9 amino acids that are otherwise conserved in bees, two of them locate in the RNP1 region. In addition, *Bombus* and *Melipona* species have one common amino acid replacement, as compared to the other species. When compared over full length, *Apis*-Tra2 shows 21 of otherwise fixed amino acid differences compared to non-*Apis* species. In previous analyses (Kapheim et al., in revision), we noticed that the RRM domain is on average more divergent between *Apis* and non-*Apis* species than outside of the domain (*P* < 0.1), predominantly for the downstream region (*P* < 0.01). These unexpected findings could hint to an *Apis* specific functional association of *tra2* with *fem*, depending on the lineage specific *fem* copies and their function.

**Figure 4:**
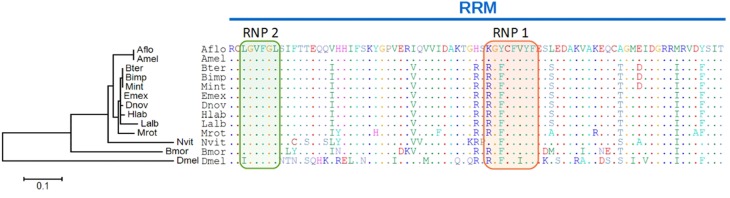
**Amino acid alignment of the tra2 RNA recognition motif and phylogenetic tree**. The RNA recognition motif (RRM) with two elements (RNP1: red, RNP2: green) known to be directly involved in RNA recognition are highlighted. The maximum likelihood tree branch length represents amino acid changes per site for *tra2*. Abbreviations are the same as for Figure 2 adding Bmor, *Bombyx mori* (Lepidoptera); Dmel, *Drosophila melanogaster* (Diptera); Mint, *Melipona interupta*.

We compared the relative evolutionary rate of *tra2* bee sequences to further evaluate the differences between *Apis* and non-*Apis* species. Using Tajima's relative rate test, we tested the null hypothesis of equal molecular clock rate between *Mint*/*Amel, Mint*/*Bter*, and *Mint*/*Bter* using four non-*Apis* species (*Mrot, Emex, Hlab, Nvit*) as outgroup. Tests on molecular evolutionary rates of *fem* reveal a higher rate in *Apis* compared to non-*Apis* species (*P* < 0.01 for all pairwise comparisons, using different outgroups). No difference in evolutionary rate was detected between non-*Apis* (*Mint*/*Bter*) *tra2* comparison (*P* > 0.5). To test, whether these evolutionary rate differences is specific to *tra2* or a general phenomenon among *Apis* and non-Apis species, two reference genes were analyzed (*elongation factor* 1 and *GB11211*—a gene know to be located in close vicinity of the *fem* gene within the sex determination locus, Hasselmann et al., 2010). No rate differences were detected between *Apis* and non-*Apis* for both genes (*P* > 0.05).

### The evolutionary conserved key regulator *doublesex*

**Figure 5:**
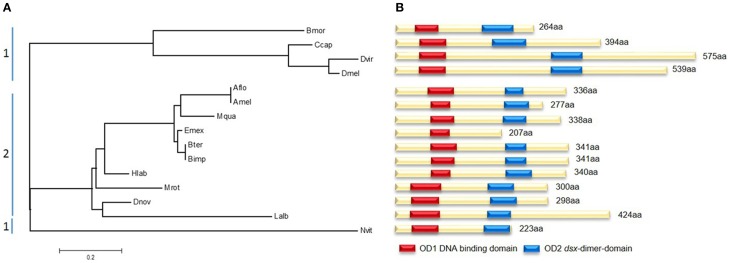
**Molecular evolutionary analysis and protein domains of *doublesex* (*dsx*)**. **(A)** Maximum likelihood tree using the JTT model represents *dsx* amino acid distances between several non-bees (1) and bees (2). Abbreviations are the same as for Figure 4 adding Ccap, *Ceratitis capitata*, Dvir, *Drosophila virilis*. According to their evolutionary relationship they are clustered in two parts. **(B)** Scheme of the different *dsx* proteins. The position of the two highly conserved domains OD1 (red) and OD2 (blue) are marked. Reduced sequence information caused the absences of OD2 in Emex.

Sequence analyses of different bee and non-bee species indicate fundamental changes in the initial regulatory elements of the sex determining pathways. Although the gene *doublesex* (*dsx*), which is located toward the bottom of the pathway, shows large amino acid sequence divergence between species, two major domains remain highly conserved (Figure 5). OD1 harbors a DNA-binding domain containing a zinc-finger, while OD2 includes a dimerization domain which was found in all analyzed species except *Eufriesea mexicana*, which could be due to poor sequence quality. The evolutionary tree of *dsx* shows a distinct segregation between bee and non-bee species (Figure 5A). This might be not only due to evolutionary distances by nucleotide changes, but also by structural changes. All non-bee species (except the wasp *Nvit*) showed a female-specific exon which was not present in the bee species. In *D. melanogaster* this exon contains six 13-nucleotide repeats, which are exonic splicing regulatory elements (ESR) and are essential for Tra2 binding to *dsx* (Baker, 1989). This repeats were not specifically found in the other non-bee species (e.g., *Bmor, Ccap*), whereas the presence of the female specific exon might suggests a similar mechanism of protein-binding to *dsx* as it was found in *Drosophila* and other dipteran species (data not shown; Ruiz et al., 2007). Since this female specific exon seems to be absent in the bee species, one hypothesis could be that these ESR are located in other positions of the gene. We tested for this and did not find any evidence of similar ESR in other positions of the gene (data not shown). Alternatively, bees might have evolved other regulatory elements transmitting the Tra2 binding to *dsx*. This hypothesis should be tested in future experimental studies.

## Discussion

Studying the evolution of genetic components within regulatory pathways may shed light on the flexibility of how similar requirements are satisfied by different approaches in nature. This ubiquitous phenomenon, known as developmental system drift (DSD) has been identified to establish homologous conserved traits by developmental mechanism that are diverged among species (True and Haag, 2001; Abouheif and Wray, 2002; Nahmad et al., 2008). Here, we focused on major regulators of the sex determination pathway in social insect species, elucidating their evolutionary dynamic. The transductional core of the sex determining pathway [*fem(tra*)/*dsx* complex] is evolutionarily conserved in insects over more than 280 million years of divergence (Diptera/Hymenoptera). Upstream initial signals regulating the sex-specific splicing of *fem/tra* may evolve within much shorter time, being consistent with the bottom-up theory (Wilkins, 1995) and the hour-glass model recently developed by Bopp et al. (2014). The different copy numbers of *fem* duplications found in bee genomes (this study and Kapheim et al., in revision) would allow either lineage specific gene loss (in *Mqua, Mrot, Dnov*, and *Hlab*) from a single ancestral duplication event or independent gene duplications (in *Apis, Bter, Bimp, Lalb*).

Our data from a variety of bee species now provide evidence for different evolutionary fates of the key regulator *fem* in bees. Gene duplications of *fem* in only some of the bee lineages in conjunction with diversifying selection seem to be the major force driving the evolution of *fem* and it paralogous copies. We identified amino acid motifs in *fem* and its copies that coincides with the prediction of protein structures (e.g., coiled-coil) known to be involved in protein interaction processes. The amino acid divergence between *Apis* and non-*Apis* species on these motifs favors the hypothesis that functional constraints may have shaped these parts of the protein differently. Among them, motif 5 reveals highest divergence between *Apis* and non-*Apis* species (73% total aa divergence, Supplementary Table 4) whereas to the low overall divergence (0.4% aa) between both groups hints to an lineage specific accumulation of amino acid changes. Recent analyses of Koch et al. (2014) provide evidence for independent gene duplication of *fem* in *Apis* and *Bombus* and reject the hypothesis of concerted evolution between *fem/csd* and *fem/fem1* as proposed by Privman et al. (2013) and Schmieder et al. (2012). By these processes, primary sex determining signals may evolve rapidly including modified function of known key regulators. This hypothesis could be supported by the greater divergence of the Tra2-RRM-domain, particularly between *Apis* and non-*Apis* bees, indicating a lineage specific functional interaction of *tra2*/*csd, tra2/fem* or *tra2/fem1*.

### Evolutionary changes in *tra2* but not in Dsx between *Apis* and non-*Apis* bees

There are several indications that *tra2* in *Apis* has evolved differently compared to other bee species. The *tra2* genealogy (Figure 4) does not match to the species phylogeny (Figure 1) derived from seven genes. The *tra2* sequences of *Apis* cluster in a separate branch from phylogenetic closely related groups and evolve with higher evolutionary rate. Reflected by the high number of *Apis*-specific amino acid changes we suggest a modified function of *tra2* compared to non-*Apis*. Changes in the amino acid composition on 21 sites, 9 of them inside the RRM-domain, led us to conclude that target molecule specificities in binding sites may have been modified. These target molecules could be *fem* and/or *dsx*. Our evolutionary analysis of Dsx protein indicates a rather high degree of structural conservation (Figure 5B). Consequently, and in agreement with the widely accepted hypothesis of bottom-up evolution in sex determining pathways (Wilkins, 1995), we have reasons to assume that *dsx* has retained its conserved function and that the structural changes in Tra2 were driven by *fem* evolution.

### Coevolutionary model of *tra2* and *fem*/paralog complex in *Apis* and non-*Apis*

The evolution of novel or modified gene function may affect the function of associated genes (Innan and Kondrashov, 2010), a characteristic that we have noticed already in the evolution of *fem* in *Apis* in a previous study (Hasselmann et al., 2010). In that study we found that *fem* in *Apis* evolves under stronger functional constraints than in non-*Apis*, likely due to the origin of the novel function raised by *csd*. Often known as coevolution, molecular changes among closely interacting genes may lead to lineage-specific modification of protein function. The concept of gene-for-gene evolution has been introduced and widely described in plant-pathogen interactions, with natural selection and genetic drift as the major evolutionary processes driving this form of coevolution (Thompson and Burdon, 1992; Dodds et al., 2006). Our present results led us to propose a model of coevolutionary changes in sex determining key regulator *tra2* and *fem* with its paralogs, depending on their presence or absence.

**Figure 6:**
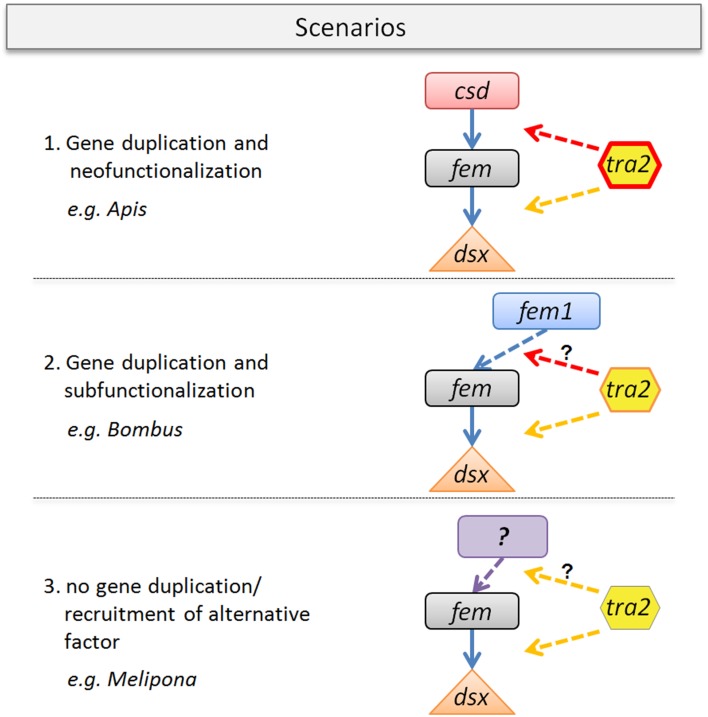
**Gene duplication-coevolutionary model for sex determining key regulators in bees**. Three possible scenarios with organismic examples are given. (1) *Fem* gene duplication that gave rise to *csd* and its neo-function in *Apis. Tra2* protein changes (red line) are specifically coevolved in conjunction of *csd* evolution, known to mediate *fem* sex-specific splicing. *Tra2* function at the *fem*/*dsx* level may have been conserved. (2) Scenario with *fem* duplication providing *fem1*-paralog (as found in *Bombus*), indicating subfunctionalization. *Tra2* changes are less pronounced which may alter the binding affinity to *fem1*. The sex determining role of fem1 need to be clarified. (3) No duplication of *fem*, as currently assumed for e.g., *Melipona* spp. *Tra2* function at the *fem*/*dsx* level is likely to be conserved whereas its possible interaction to the so far unknown primary signal of sex determination requires further investigation.

We propose three scenarios that may impact the evolution of the *tra2*/*fem*/paralog gene complex. Scenario one resembles the best studied case so far (Figure 6) found to be established in the *Apis* lineage. In this scenario, the evolution of the multiallelic *csd* operating as primary signal of sex determination following the model of neofunctionalization was accompanied by lineage specific changes of the Tra2 protein. Tra2 has been proven to mediate *fem* mRNA sex-specific splicing, transmitting the information of the allelic composition at *csd* to its downstream target (Nissen et al., 2012). Consequently, our data of numerous *Apis*-specific amino acid substitutions (Figure 4) within and outside of the RRM domain indicates a coevolutionary, fast-evolving process forced by the strong directional evolution that has acted on *csd* (Hasselmann et al., 2008). In addition, Tra2 has been proposed to interact with the genes *fem* and *dsx* to act on regulating sex-specific splicing of *dsx* (Nissen et al., 2012). To disentangle which of the amino acid changes are directly associated to this twofold functions of *tra2*, future *in vitro* studies are needed.

The second scenario illustrates duplication events of *fem*, as found in e.g., *Bombus*, giving rise to the paralogous copy *fem1* (Sadd et al., in press). The proposed model of subfunctionalization (Figure 6) is supported by the absence of allelic variation in *fem1* which is in contrast to the *fem* paralog in *Apis* (*csd*) (Biewer et al., in revision). Another difference between *csd* and *fem1* is the occurrence of various splice transcripts in the latter and their absence in *csd* (Gempe et al., 2009). We hypothesize that the numerous amino acid differences in Tra2 are associated with its modified binding specificity in *Bombus* (dotted arrow), driven by a different evolutionary fate of the *fem* paralog. Still, it remains up to further investigation to identify the primary signal of sex determination in *Bombus* and the position of *fem1* within the sex determination pathway.

Our last scenario three (Figure 6) is stimulated by the observation that in some bee species (e.g., *Melipona*) obviously no *fem* duplication exist. This result is not only supported by bioinformatic approaches on newly sequenced genome data (this study and Kapheim et al., in revision) but also by various experimental setups (Brito et al., unpublished). In this scenario *tra2* function is likely to be related to the sex determination pathway based on its evolutionary conservation (this study) and on its constant expression over early (egg) and late (larvae, pupae, adults) developmental stages in *Melipona interupta* (Schlesinger and Hasselmann, unpublished data). Gene expression studies can add another useful dimension to examine coevolution among genes as interacting proteins are often precisely coexpressed, (Fraser et al., 2004), ultimately leading to a better understanding of protein interaction processes within regulatory pathways. Further, analyses will likely elucidate the primary signal of sex determination in *Melipona*, a system on which various alternative models to explain the determination of different sexes have been developed in the past, including empirical evidence for a complementary mode of sex determination resulting from controlled crossing experiments (Kerr, 1987; Carvalho, 2001).

Our comparative analyses of major regulators of sex determination in hymenopteran species provide further support to the wide range of evolutionary possibilities for shaping the sex determination regulatory pathway, consistent with the concept of DSD. Driving forces affecting the evolutionary dynamic of sex determining key regulators are gene duplication, selection and coevolution. More instantaneously occurring events such as transposon mediated translocation of genes or fragments and recombination events may lead to gene copy number variations, including pseudogenization (Lonnig and Saedler, 2002). These processes are likely to be common in hymenopteran species, as high recombination frequencies in bees and ants (Beye et al., 2006; Sirvio et al., 2006; Meznar et al., 2010) and transposable elements near sex determining genes (Beye et al., 2003; Koch et al., 2014) have been observed. For the hymenopteran wasp species *Nasonia vitripennis* a non-complementary sex determining system has been recently proposed, based on maternal effected genomic imprinting (van de Zande and Verhulst, 2014). To ensure male development in unfertilized eggs, a *womanizer* factor, which is maternally silenced during oogenesis and affects *tra* expression, has been described, opening the road to study the probably highly divergent alternative mechanism that has evolved in course of wasp and bee divergence. The challenge for future studies on species with newly sequenced genomes will be to test evolutionary predictions raised by bioinformatic analyses using functional experiments.

### Conflict of interest statement

The authors declare that the research was conducted in the absence of any commercial or financial relationships that could be construed as a potential conflict of interest.
