# Supplementary material for: The evolutionary dynamics of major regulators for sexual development among Hymenoptera species
Source: Front Genet. 2015 Apr 10;6:124. doi: 10.3389/fgene.2015.00124 (PMC4392698; doi:10.3389/fgene.2015.00124)
Supplement: Supplementary file 1 [file Table1.PDF]

Supplementary Material

The evolutionary dynamics of major regulators for sexual development among Hymenoptera species

Matthias Biewer[1,2], Francisca Schlesinger[1,3], Martin Hasselmann[1,2]*

[1]Population Genetics of Social Insects, Institute of Genetics, University of Cologne, Cologne, Germany
[2]Livestock Population Genomics Group, Institute of Animal Science, University of Hohenheim, Stuttgart, Germany
[3]Institute of Bee Research, Hohen Neuendorf, Germany

*** Correspondence:** Prof. Dr. Martin Hasselmann, Livestock Population Genomics Group, Institute of Animal Science, University of Hohenheim, Garbenstrasse 17, Stuttgart, 70599, Germany
martin.hasselmann@uni-hohenheim.de

1. Supplementary Figures and Tables

1.1. Suplementary Tables

Supplementary Table 1. Data set used for the molecular evolutionary analysis of *fem/tra* copies

Gene	Species (ID)	OrthoDB/Kapheim et al.	GenBank
fem	Aflo02267	x	
	Amell		EU100941
	Acer		EU100936
	Adors		EU100938
csd	Aflo02266	x	
	Amell		EU100892
	Adors		EU100932
	Acer		EU100907
fem1	Bter		XM_003394645
fem	Bter		NM_001280924
fem	Bimp		XM_003493748
fem1	Bimp14017	x	
fem	Dnov10858	x	
fem	Emex02197	x	
fem	Hlab07879	x	
fem	Lalb_10095	x	
fem1	Lalb_10228	x	
fem	Mrot08431	x	
fem	Mqua06253	x	
tra	Nvitr		NM_001134827
		**Privman et al. (2013)**	
tra	Aech_tra	x	
tra	Acep_traA	x	
tra	Acep_traB	x	
tra	Pbar_traA	x	
tra	Pbar_traB	x	
tra	Cflo_traA	x	
tra	Cflo_traB	x	
tra	Lhum_traA	x	
tra	Lhum_traB	x	
tra	Hsal_traA	x	
tra	Hsal_traB	x	
tra	Sinv_traA	x	
tra	Sinv traB	x	

Supplementary Table 2. Data set used for the molecular evolutionary analysis of *dsx* copies

species	OrthoDB/Kapheim et al.	GenBank
Apis florea	Aflo03380	
Apis mellifera female		EU236957
Bombus terrestris	Bter02495	
Bombus impatiens	Bimp05107	
Dufourea novaeangliae	Dnov10920	
Eufriesea mexicana	Emex00223	
Habropoda laboriosa	Hlab01811	
Lasioglossum albipes	Lalb_01555	
Megachile rotundata	Mrot01361	
Melipona quadrifasciata	Mqua08015	
Ceratitis capitata		AF435087
Drosophila melanogaster		NM_169202.1
Nasonia vitripennis		NM_001162517.1
Drosophila virillis		XM_002056562.1
Bombyx mori		NM_001043406.1

Supplementary Table 3. Data set used for the molecular evolutionary analysis of *tra2* copies

species	OrthoDB/Kapheim et al.	GenBank
Apis florea	Aflo04333	
Apis mellifera		GB47305
Bombus terrestris		XM_003398958.1
Bombus impatiens		XM_003485456.1
Dufourea novaengliae	Dnov04996	
Eufriesea mexicana	Emex10063	
Lasioglossum albipes	Labl_13632	
Megachile rotundata	Mrot01874	
Melipona quadrifasciata	Mqua09093	
Habropoda laboriosa	Hlab07817	
Nasonia vitripennis		XM_001601056
Drosophila melanogaster		FBgn23633
Bombyx mori		NM_001126233

Supplementary Table 4. Amino acid divergence of *fem* (and paralogs) motifs between groups of *Apis* and non-*Apis* bees

	Apis / non-Apis		Within	
	net divergence	total divergence	Apis	non-Apis
Motif 1	0.134 +/- 0.045	0.354 +/-0.084	0.115 +/-0.03	0.325 +/-0.063
Motif 2	0.065 +/- 0.068	0.23 +/- 0.068	0.131 +/- 0.049	0.199 +/- 0.063
Motif 3	0.121 +/- 0.048	0.384 +/-0.078	0.166 +/- 0.045	0.360 +/- 0.075
Motif 4	0.033 +/-0.026	0.272 +/- 0.069	0.178 +/- 0.064	0.301 +/- 0.112
Motif 5	0.044 +/-0.166	0.731 +/- 0.184	0.224 +/-0.057	0.357 +/- 0.082
Motif 6	0.295 +/-0.162	0.606 +/- 0.177	0.372 +/- 0.110	0.250 +/- 0.078

Note: Apis groups are represented by *A. mellifera*, *A. cerana*, *A. dorsata* and *A. florea*, non-*Apis* bees by *B. terrestris*, *B. impatiens*, *E. mexicana*, *M. quadrifasciata*, *H. laboriosa*, *M. rotundata*, *D. novaeangliae* and *L. albipes*. Amino acid substitutions per site were calculated using the JTT-matrix based model, as net average between groups and from averaging over all sequence pairs between group (total divergence) using software MEGA6.
