# Supplementary material for: The evolutionary dynamics of major regulators for sexual development among Hymenoptera species
Source: Front Genet. 2015 Apr 10;6:124. doi: 10.3389/fgene.2015.00124 (PMC4392698; doi:10.3389/fgene.2015.00124)
Supplement: Supplementary file 2 [file Image1.PDF]

Supplementary Material

The evolutionary dynamics of major regulators for sexual development among Hymenoptera species

Matthias Biewer[1,2], Francisca Schlesinger[1,3], Martin Hasselmann[1,2]*
[1]Population Genetics of Social Insects, Institute of Genetics, University of Cologne, Cologne, Germany
[2]Livestock Population Genomics Group, Institute of Animal Science, University of Hohenheim, Stuttgart, Germany
[3]Institute of Bee Research, Hohen Neuendorf, Germany
Correspondence: Prof. Dr. Martin Hasselmann, Livestock Population Genomics Group, Institute of Animal Science, University of Hohenheim, Garbestrasse 17, Stuttgart, 70599, Germany
martin.hasselmann@uni-hohenheim.de

1. Supplementary Figures and Tables

1.2. Supplementary Figures

Supplementary Figure 1

Sequence logos of amino acid motifs and their phylogenetic signal of *fem* and paralogous copies. Conserved motifs were identified using the MEME package (see Material and Methods) and maximum likelihood trees represent amino acid per site divergence.

motif 2

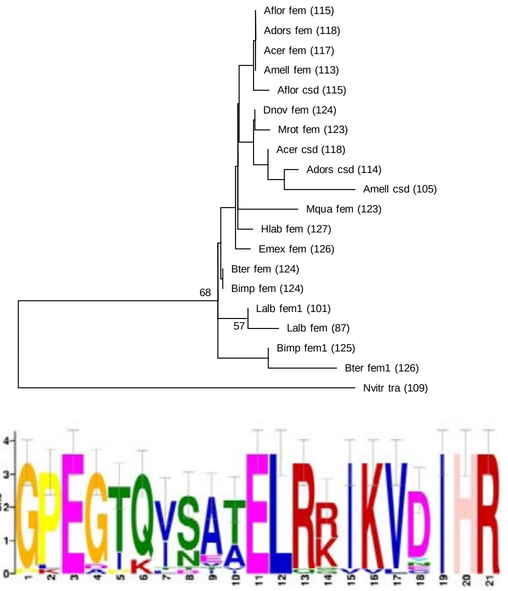

motif 3

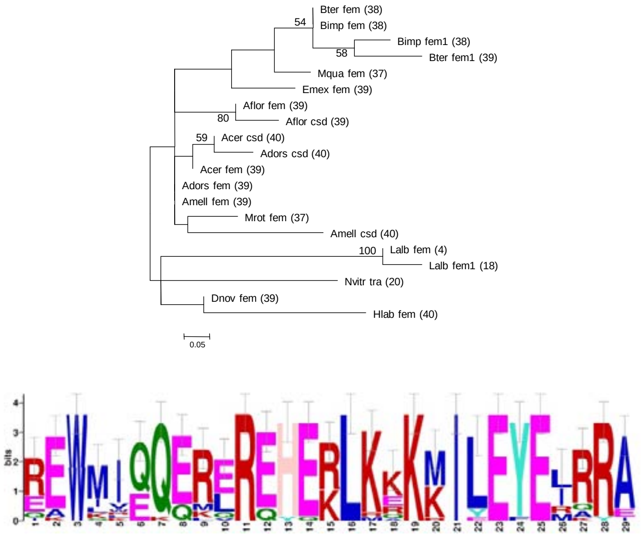

motif 4

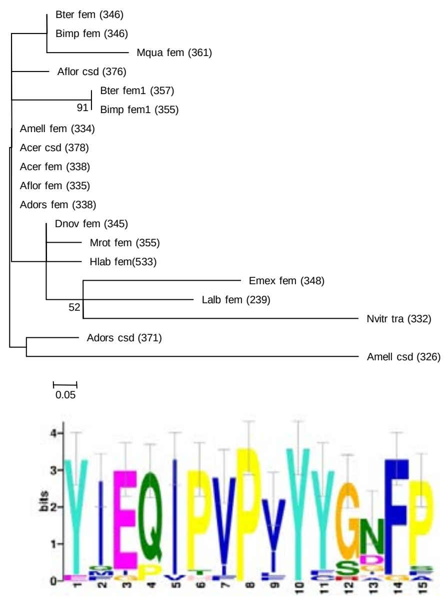

Supplementary Figure 2

Relative position of amino acid motifs in fem of bee species .
Abbreviations as described in Fig.2. The combined best matches of a sequence to a group of motifs are given (combined p-values).

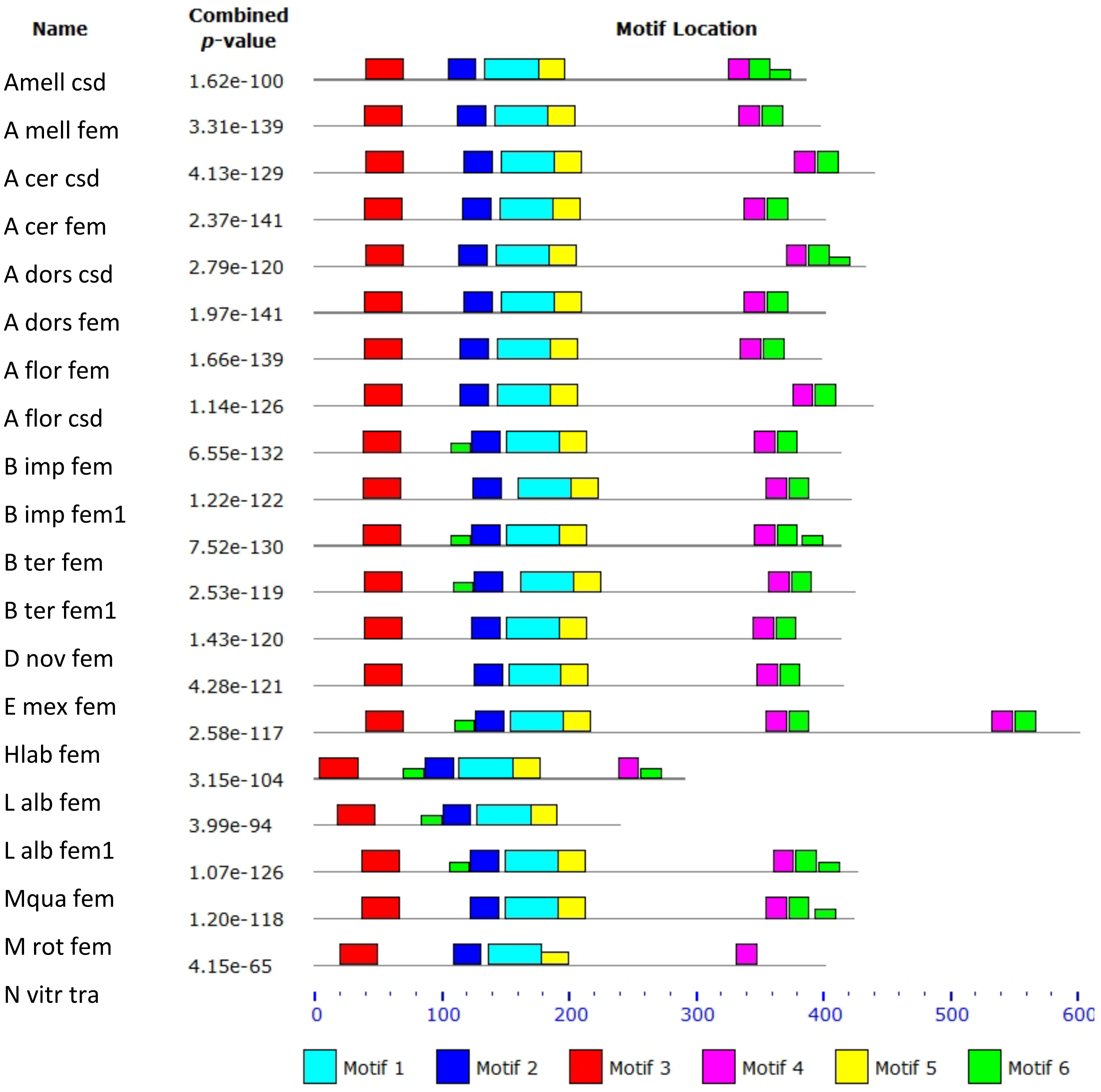

Supplementary Figure 3

Motif evolution in fem paralogs of bees. Homologous amino acid sequences of *fem*, *csd* and *fem1* in bees in the region for which an coiled-coil motif is predicted in *csd-Amel*, *csd-Adors* and *csd-Acer* (relevant amino acids are marked with *)

```
                 *          **     **  *
csd  Amel   CSRDRNREYRKKDRRYEKLY--NEKEKLLEERTSRKRYSR
csd  Ador   .........K....Q..............Q.K...--...
csd  Acer   ..G...K..K....Q....RTD......SQ.K........
csd  Aflor  .......G.K....Q........V.....R.K..L.....
fem  Amel   .....S...K......DQ.H...VE..H.R.....R....
fem  Acer   .....S...K......DQ.H...VE..H.R.....R....
fem  Ador   .....S...K......DQ.H...VE..H.R...N.R....
fem  Aflor  .....S...K.-N....P.H...VE..H.R.....R....
fem  Bter   H....S...KETVK.HSQ.N..TAE.EH.R..KGHRDH..
fem1 Bter   H..GGS...KE.V.PHSQ.H..TAE..H.RK.K.HRDN.H
fem1 Bimp   H..GGS...KE.V.LHSQ.H..TAE..H.R..K.NRDN.H
fem  Bimp   H..G.S...KETV..HSQ.N..TAE.EH.R..KGHRDH..
fem  Mint   R..G.S...KE....HSQ.H..TTR.----..KGHRDH..
fem  Dnov   K..EYKDSC.EE...HVHSH..SLEDERNR--KG.RDH..
fem  Emex   R..GKSK..QE....HGESR..DVE.DH.R..K.HQDH..
fem  Hlab   R..KYRES.KE.G..QPHDV..K.QHLRER--K..RD...
fem  Mrot   K..EYREC.TE...VHTHPH..GVEKDRSR..R..REH..
fem  Ehem   R..GKSK..QE....HGESR..DIE.DH.R..K.HRDH.H
```
